# Hypothalamic-Pituitary-Ovarian Axis Reactivation by Kisspeptin-10 in Hyperprolactinemic Women With Chronic Amenorrhea

**DOI:** 10.1210/js.2017-00328

**Published:** 2017-10-16

**Authors:** Robert P. Millar, Charlotte Sonigo, Richard A. Anderson, Jyothis George, Luigi Maione, Sylvie Brailly-Tabard, Philippe Chanson, Nadine Binart, Jacques Young

**Affiliations:** 1 Centre for Neuroendocrinology, Departments of Immunology and Physiology, Faculty of Health Sciences, University of Pretoria, Pretoria 0084, South Africa; 2 Institute for Infectious Diseases and Molecular Medicine, University of Cape Town, Observatory 7925, South Africa; 3 INSERM U1185, Paris Sud Medical School, Le Kremlin-Bicêtre, F-94276, France; 4 Reproductive Endocrinology Department, Le Kremlin-Bicêtre, F-94275, France; 5 MRC Centre for Reproductive Health, Queens Medical Research Institute, University of Edinburgh, Edinburgh EH16 4TJ, United Kingdom; 6 Assistance Publique Hôpitaux de Paris (AP-HP), Bicêtre Hospital, Hormonology and Genetic Department, Le Kremlin-Bicêtre, F-94275, France; 7 Université Paris Sud, Paris-Sud Medical School, Le Kremlin-Bicêtre, F-94276, France

**Keywords:** GnRH, hyperprolactinemia, hypogonadotropic hypogonadism, kisspeptin, luteinizing hormone, neurokinin B

## Abstract

**Context:**

Hyperprolactinemia-induced hypogonadotropic amenorrhea (hPRL-HA) is a major cause of hypothalamic gonadotrophin-releasing hormone (GnRH) deficiency in women. In hyperprolactinemic mice, we previously demonstrated that hypothalamic kisspeptin (Kp) expression was diminished and that Kp administration restored hypothalamic GnRH release, gonadotropin secretion, and ovarian cyclicity, suggesting that Kp neurons could also play a role in hPRL-HA.

**Objective:**

To study the effect of Kp-10 on the gonadotropic-ovarian axis in women with hPRL-HA.

**Patients:**

Two women (32 and 36 years old) with chronic hPRL-HA (prolactin: between 94 and 102 and 98 and 112 ng/mL, respectively) caused by cabergoline-resistant microprolactinomas.

**Interventions:**

Cabergoline was discontinued 6 months before inclusion. Blood samples were taken every 10 minutes for 12 hours during 2 consecutive days to evaluate luteinizing hormone (LH) and follicle-stimulating hormone (FSH) secretion. Serum estradiol (E2), testosterone (T), and inhibin B (IB) levels were also measured. Vehicle or Kp-10 (1.5 μg/kg/h) was infused intravenously for 12 hours.

**Results:**

Kp-10 induced a significant increase in LH and FSH levels and increased LH pulses. E2, T, and IB serum levels were also significantly increased.

**Conclusions:**

In this exploratory study, we demonstrated that administration of Kp-10 reactivated gonadotropin secretion in women with hPRL-HA and increased ovarian activity. Our data suggest that, as in rodents, GnRH deficiency in hPRL-HA is also mediated by an impairment of hypothalamic Kp secretion. Kp-10 or its analogues could have therapeutic application as an alternative approach to restore ovarian function and fertility in women with hPRL-HA resistant to dopamine agonists and in whom pituitary surgery is not possible.

## Teaser

Diminished LH, FSH, estradiol, and inhibin B are restored by kisspeptin in women with hyperprolactinemic amenorrhea.

Hyperprolactinemia (hPRL) is a well-established and frequent cause of hypogonadotropic hypogonadism in women and men and is one of the main etiologies of anovulatory infertility in premenopausal women [1–5]. However, the precise mechanism whereby prolactin inhibits pituitary gonadotropin secretion is still unclear in humans [1, 2].

The combined luteinizing hormone (LH) and follicle-stimulating hormone (FSH) deficiencies associated with hPRL were first proposed to result from a direct effect of prolactin (PRL) on gonadotrophin-releasing hormone (GnRH) neurons, leading to a decrease in GnRH release [1, 2, 6]. This hypothesis was supported by the demonstration that pulsatile GnRH replacement reversed both hypogonadotropic hypogonadism and infertility in women and men with hPRL [7–9]. This further suggested that PRL excess in humans affects hypothalamic release of GnRH rather than directly affecting pituitary or gonadal function [1, 2, 6–9].

An unresolved question is whether elevated PRL directly affects GnRH neurons via PRL receptors (PRLRs) or indirectly affects GnRH neurons by acting upstream on other intermediate neurons that regulate GnRH secretion. Because very few GnRH neurons express PRLRs [10–13], the latter suggestion appears more likely. It is now well established that GnRH neurons are stimulated by kisspeptin (Kp) neurons and that Kp neurons unequivocally express PRLRs [14, 15]. Furthermore, we demonstrated in a female mouse model of hypogonadotropic anovulation induced by hPRL that hypothalamic Kp expression was diminished but that of neurokinin B (NKB) was not and that peripheral Kp administration could restore GnRH and gonadotropin secretion and ovarian cyclicity [16]. These results as well as the observation of reduced hypothalamic Kp expression during lactation-induced hPRL in mice strongly supported the notion that Kp plays a role in mediating hPRL-induced anovulation in mice [11, 12]. In the current exploratory study, we explored whether diminished Kp might underlie hPRL-induced hypogonadotropic amenorrhea (hPRL-HA) in women. To shed light on this, we determined whether Kp administration could reactivate the gonadotropic axis in women with hPRL-HA.

## 1. Patients and Methods

Two premenopausal women with amenorrhea caused by PRL microadenomas were included in this exploratory study. Patients 1 and 2 were 32- and 36-year-old women with 6 to 11 months of secondary amenorrhea and galactorrhea. Serum PRL levels ranged from 94 to 102 ng/mL and from 98 to 112 ng/mL in patient 1 and patient 2, respectively (normal range, 9.0 to 20 ng/mL). Estradiol (E2) levels were low, and serum gonadotropins were low or inappropriately normal, indicating hPRL-HA (Table 1) [1, 2]. Pituitary magnetic resonance imaging revealed microadenomas that were 5 and 6 mm in diameter in patients 1 and 2, respectively. Neither of these patients had other pituitary hormone deficiencies or excesses at diagnosis (normal free T4, thyroid stimulating hormone, insulin-like growth factor-1, normal cortisol and growth hormone responses to hypoglycemic challenge, and normal urinary free cortisol) (Table 1). Administration of cabergoline at doses of 2 to 3 mg per week for 5 and 7 months in patients 1 and 2, respectively, did not result in a decrease in circulating PRL concentrations, which were 100 and 102 ng/mL, or a decrease in prolactinoma size, which was 5 mm and 6.5 mm, respectively, after treatment. Cabergoline was discontinued because of a failure to respond 6 months before patient inclusion. At the time of the study, neither patient was taking any medication. No menstrual bleeding had occurred during or after cabergoline discontinuation, although galactorrhea persisted.

**Table 1.** **Main Features of Two Patients With Prolactin-Secreting Microadenomas at Diagnosis**

	**Patient 1 (P1)**	**Patient 2 (P2)**	**Normal Range**
Age, y	32	36	—
Menstrual cycle	Amenorrhea*^a^*	Amenorrhea*^b^*	Regular (27–32 days)
Galactorrhea	Yes	Yes	—
Microadenoma at pituitary MRI	5-mm diameter intrasellar	6-mm diameter intrasellar	—
Prolactin, ng/mL	94–102*^c^*	98–112*^c^*	9–20
FSH, IU/L	4.2	4.9	(3.1–7.6)*^d^*
LH, IU/L	3.9	2.1	(2.9–7.8)*^d^*
Estradiol, pg/mL	21	17	(22–97)*^d^*
Progesterone, ng/mL	0.2	0.3	(4.9–14.4)*^e^*
Inhibin B, pg/mL	49	28	(29–126)*^d^*
Cortisol, ng/mL*^e^*	108/265*^f^*	124/209*^f^*	(89–220)/(198–336)*^f^*
Free T4 (pmol/L)/TSH (mIU/L)	16.8/2.7	15.2/1.9	(10.4–21)/(0.6–4.4)
IGF1, µg/L	273	232	(169–311)*^g^*
DHEAS, ng/mL	1954	1386	(679–3993)*^g^*

To convert estradiol in pg/mL to pmol/L, multiply by 3.671. To convert the prolactin values in ng/mL to mIU/L, multiply by 21.3. For DHEAS, to convert ng/mL to nmol/L, multiply by 2.714.

Abbreviations: DHEAS, dehydroepiandrosterone sulfate; FT4, free thyroxine; IGF1, insulin-like growth factor-1; MRI, magnetic resonance imaging; TSH, thyroid stimulating hormone.

^a^ Since 6 months.

^b^ Since 11 months.

^c^ Range (different measures and after exclusion of macroprolactinemia).

^d^ In the early follicular phase.

^e^ In the normal luteal postovulatory phase.

^f^ Basal/peak under hypoglycemia challenge test.

^g^ In healthy 30- to 40-year-old women.

### A. Protocol

Kp-10 was custom-synthesized under Good Manufacturing Practices standards (Bachem GmbH, Weil am Rhein, Germany) and aliquoted in vials as described previously [17–20]. The lyophilized Kp-10 was reconstituted in 5 mL of sterile physiological saline as previously described [17]. The Paris Sud University and Bicêtre Hospital ethics committees approved the study (CPP Ile-de-France VII, Bicêtre Hospital, and ANSM ID RCB: 2017-A02140-53), and the two participants gave their informed consent. Subjects were admitted to the hospital at 08:00 hours for 12 hours of blood sampling every 10 minutes for 2 consecutive days as previously described [17]. Vehicle (physiological saline) or Kp-10 in saline (1.5 µg/kg/h) was infused intravenously from 08:30 to 20:30 hours on days 1 and 2, respectively; randomization was not performed because of the possibility of Kp-10−induced activity confounding results during vehicle infusion. There were no adverse events related to Kp-10 infusion, as previously reported [17–20].

### B. Hormone Assays

Serum PRL level was measured by immunofluorescence using Time Resolved Amplified Cryptate Emission (TRACE™) technology with a Brahms KRYPTOR™ platform (Thermo Fisher, Hennigsdorf, Germany). The detection limit was 0.1 ng/mL. The intra-assay coefficient of variation (CV) was 3.5%. In both patients, sera were treated with polyethylene glycol to exclude measurement of macroprolactin [21]. Serum FSH and LH levels were measured using sensitive immunoradiometric assays (CIS bio international, GIF sur Yvette, France) as reported [22], with a detection limit of 0.05 IU/L for both hormones (IU/L, second International Reference Preaparation World Health Organization 78/549 for FSH; IU/L, first IRP 68/40 for LH). The intra-assay CV was 1.5% for LH and 2.7% for FSH using quality control sera measuring 3.5 and 3.9 IU/L, respectively [22]. Serum inhibin B (IB) levels were measured with the Beckman Coulter Gen II assay (Marseilles, France), with a detection limit of 3 pg/mL and intra-assay CVs of 6% at 15 pg/mL [22]. Serum E2 and testosterone were assayed in a single batch in all samples using gas chromatography/mass spectrometry, as described in detail by Giton *et al.* [23]. The lower limits of detection and the intra-assay CV were 2.4 pg/mL and 7.9%, respectively, for E2 using quality control serum. The lower limits of detection and the intra-assay CV were 0.05 ng/mL and 2.2% for testosterone (T).

### C. Statistical Analysis

Pulses were detected by the Thomas algorithm by analysis of LH concentrations in samples collected at 10-minute intervals [24, 25]. Mean serum LH and FSH levels during infusion of vehicle (n = 72 measurements of each gonadotropin) or Kp-10 (n = 72 measurements of each gonadotropin) were compared in each of the patients with the paired nonparametric Wilcoxon test. In the two patients, mean serum IB levels (n = 12 measurements during each treatment) and mean serum E2 levels (n = 12) were compared with the paired nonparametric Wilcoxon test. *P* values <0.05 were considered statistically significant.

## 2. Results

Kp-10 was well tolerated, and no adverse side effects were found in this study as in previous studies using this regimen of dose and treatment duration [17–20].

### A. Gonadotropins

**Figure 1.**
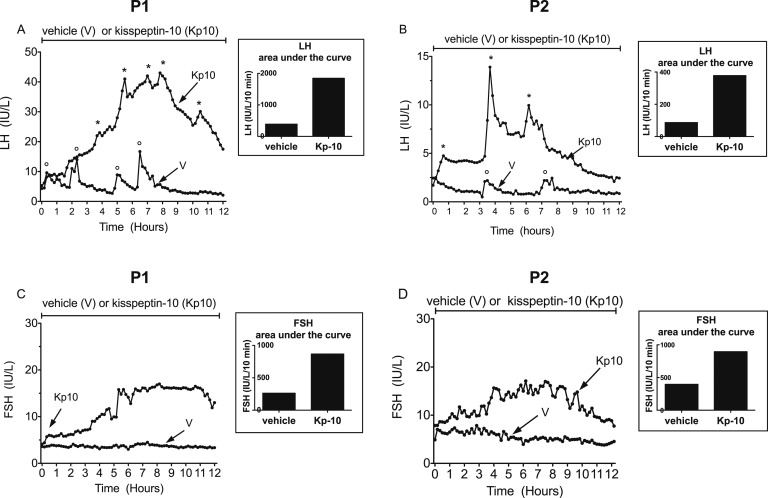
Secretory pattern of LH and FSH during infusion of saline (vehicle) or kisspeptin-10 (Kp-10) in two female patients (P1 and P2) with hyperprolactinemia-induced hypogonadotropic amenorrhea. Asterisks and open circles denote LH pulses as identified by the Thomas algorithm while the patients were receiving Kp-10 and vehicle, respectively [24, 25]. See also Table 2. During vehicle administration, serum prolactin levels were between 108 and 111 ng/mL in P1 and between 112 and 129 ng/mL in P2. During Kp-10 infusion, serum prolactin levels were between 103 and 114 ng/mL in P1 and between 116 and 120 ng/mL in P2. Area under the curve values are depicted in the histograms.

Circulating LH and FSH concentrations and pulsatility during 12-hour vehicle or Kp-10 (1.5 µg/kg/h) infusions in patients 1 and 2 are shown in Fig. 1. A significant increase in circulating LH levels was observed in both patients during Kp-10 infusion. In patient 1, four significant LH pulses were detected during vehicle administration by the Thomas algorithm [24, 25], whereas five were detected during Kp-10 infusion (Fig. 1A). In this patient, mean serum LH levels increased from a mean ± standard deviation of 5.3 ± 2.9 IU/L during vehicle administration to 25.4 ± 10.8 IU/L (*P* < 0.0001) during Kp-10 infusion (Table 2). In patient 2, two significant LH pulses were detected during vehicle infusion vs three during Kp-10 administration (Fig. 1B), and mean serum LH levels increased significantly (*P* < 0.0001) from 1.22 ± 0.43 IU/L during vehicle infusion to 5.2 ± 2.4 IU/L during Kp-10 infusion (Table 2). Circulating FSH concentrations were also significantly higher (*P* < 0.0001) in the two patients during Kp-10 infusion than during vehicle infusion (Fig. 1C and 1D; Table 2).

**Table 2.** **LH, FSH, Estradiol, Testosterone, and Inhibin B Concentrations in Women With Hyperprolactinemia Receiving Vehicle (Saline) and Then Kp-10 Infusion**

	**Patient 1 (P1)**		**Patient 2 (P2)**		**Normal Range*****^a^***
	**Saline**	**Kp-10**	**Saline**	**Kp-10**	
LH, IU/L	5.3 ± 2.9 (n = 72)	25.4 ± 10.8*^b^* (n = 72)	1.22 ± 0.43 (n = 72)	5.2 ± 2.37*^b^* (n = 72)	2.2–8.2
FSH, IU/L	3.6 ± 0.3 (n = 72)	12.1 ± 4.3*^b^* (n = 72)	5.5 ± 1.0 (n = 72)	12.2 ± 2.7*^b^* (n = 72)	2.4–7.9
Estradiol, pg/mL	18.6 ± 4.5 (n = 12)	47.5 ± 20.9*^c^* (n = 12)	16.8 ± 1.9 (n = 12)	24.7 ± 8.6*^d^* (n = 12)	18–97
Testosterone, ng/mL	0.78 ± 0.04 (n = 12)	0.92 ± 0.08*^c^* (n = 12)	0.62 ± 0.02 (n = 12)	0.69 ± 0.02*^c^* (n = 12)	0.3–0.8
Inhibin B, pg/mL	50.5 ± 5.7 (n = 12)	188.0 ± 69.4*^b^* (n = 12)	28 ± 2.4 (n = 12)	40.9 ± 6.4*^c^* (n = 12)	29–129

Data are presented as mean ± standard deviation. During saline control and Kp-10, 12 measurements of estradiol and inhibin B were performed. To convert estradiol in pg/mL to pmol/L, multiply by 3.671. To convert testosterone values in ng/mL to nmol/L, multiply by 3.467.

^a^ Normal values were taken from the early follicular phase, days 3 to 6 after onset of menses; data from 51 healthy women [22].

^b^ *P* < 0.0001 (Wilcoxon test).

^c^ *P* < 0.01 (Wilcoxon test).

^d^ *P* < 0.05 (Wilcoxon test).

### B. Ovarian Hormones

**Figure 2.**
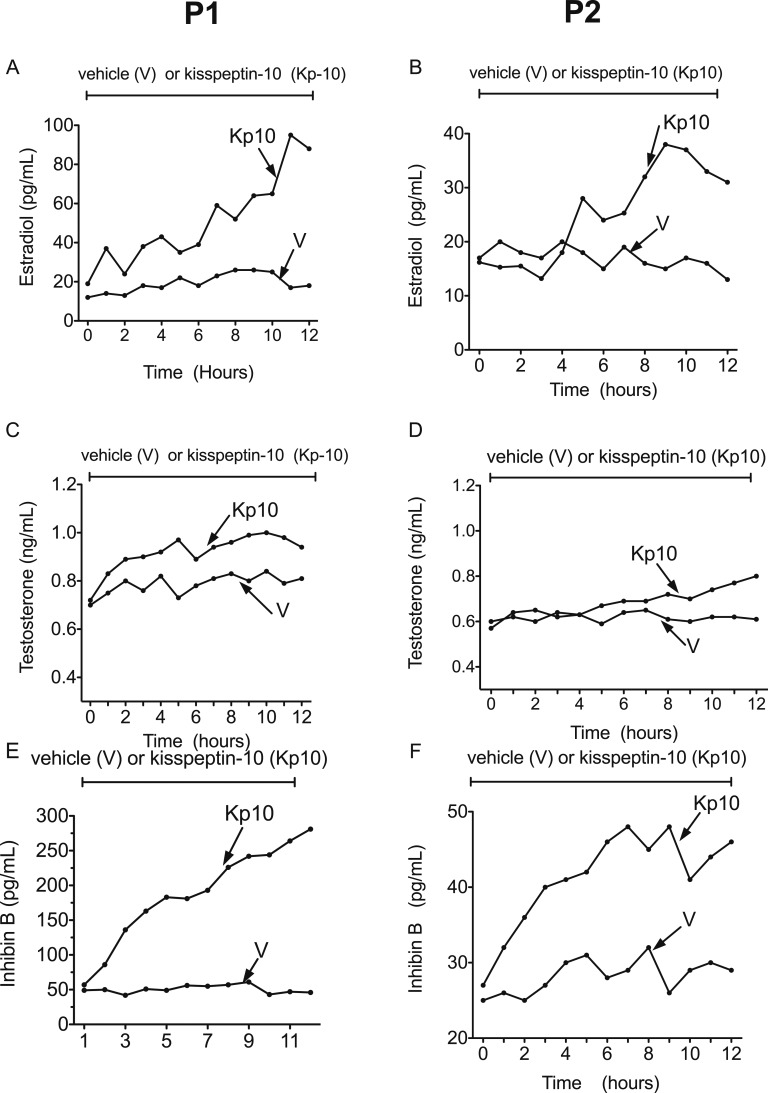
Effect of vehicle or Kp-10 administration on circulating estradiol, testosterone, and inhibin B levels in the two patients (P1 and P2) with hyperprolactinemia-induced hypogonadotropic amenorrhea. See also Table 2.

Circulating E2, T, and IB concentrations during 12-hour vehicle or Kp-10 (1.5 µg/kg/h) infusions in patients 1 and 2 were significantly increased during Kp-10 infusion (Fig. 2; Table 2).

## 3. Discussion

hPRL is a common cause of hypogonadotropic hypogonadism in men and women and one of the leading causes of anovulatory infertility (WHO Group I) in premenopausal women [1–5]. However, the precise mechanism underlying the association has not been fully elucidated. The proposal that LH and FSH deficiencies associated with hPRL are mediated by a direct inhibitory effect of PRL on GnRH neurons was supported by the demonstration that recombinant PRL administration suppressed pulsatile endogenous LH secretion in healthy women [6] and that pulsatile GnRH replacement can reverse hypogonadotropic hypogonadism and restore fertility in women and men with hPRL [7–9]. This observation also suggests that PRL effects are not directly on the pituitary or gonads. Although the findings indicated that PRL effects were mediated by an inhibition of GnRH secretion, they did not resolve whether elevated PRL level directly affects the GnRH neurons or whether this action is mediated by an inhibition of upstream neurons, such as Kp and NKB, which stimulate GnRH secretion [1, 16, 17]. The latter possibilities appeared more likely because very few GnRH neurons expressed PRLRs [10, 14]. On the other hand, Kp neurons, which also express NKB in the arcuate nucleus, were particularly cogent targets for hPRL effects, as they robustly expressed PRLRs in laboratory rodents [10]. Moreover, we demonstrated that in a female mouse model of hypogonadotropic anovulation induced by continuous infusion of PRL, hypothalamic Kp messenger RNA and protein were diminished, whereas there was no effect on NKB encoding gene expression [16]. Furthermore, peripheral Kp-10 administration restored GnRH and gonadotropin secretion and ovarian cyclicity [16]. We therefore reasoned that the same mechanism might underlie hPRL hypogonadotropic hypogonadism in humans and that Kp-10 administration would restore gonadotropin levels and ovarian activity.

To examine this proposal, we recruited two women with lactotrope microadenomas that were resistant to cabergoline therapy and who had chronically elevated PRL levels and secondary amenorrhea with galactorrhea, reduced or inappropriately normal gonadotropins, and low E2 levels. Both patients showed robust elevations of LH, FSH, E2, and IB. Ideally, we would have included more subjects in the study, but these patients are difficult to recruit because resistance to cabergoline therapy (*i.e.,* failure to normalize PRL levels) in women with PRL microadenomas is rare [2, 26, 27]. We therefore felt that the results were sufficiently clear-cut to advance knowledge in the area.

Although Kp-10 robustly increased serum concentrations of gonadotropins, E2, T, and IB, it is uncertain that this would be maintained in chronic administration. We have shown in mice exposed to continuous PRL that ovarian cycles and oocyte development can be restored by single daily doses of Kp-10 [16], suggesting the treatment will be effective in patients with hPRL. This suggestion is supported by the observation that twice-daily administration of Kp-54 did not affect menstrual cyclicity and did not abolish acute stimulation of LH after injection of Kp-54 or GnRH [28].

In both patients, the rise in LH and FSH levels continued for about 8 hours of Kp-10 infusion; however, thereafter both gonadotropins declined but were still considerably higher than levels during vehicle infusion. This decline could be due to desensitization to Kp-10 (tachyphylaxis). However, we and others have not seen desensitization in humans over this time frame [17], even after 32 hours of continuous infusion at this dose [19]. It is therefore more likely that the rising E2 and IB levels were responsible for this decline in FSH and LH values through physiological negative feedback. It is also pertinent that the current study used continuous infusion, whereas in the therapeutic setting, single daily doses would be administered as was efficacious in our mouse studies. Moreover, as mentioned earlier, in healthy women twice-daily injection of Kp for 5 days did not abolish acute stimulation of LH after injection of Kp-54 [28]. A similar protocol would be used for women with hPRL and the reasonable expectation of follicular development and ovulation. Because elevated PRL levels in our mouse model decreased Kp gene and protein expression without affecting NKB, the use of Kp-10 to restore fertility to hPRL would appear to be more physiological than use of GnRH or gonadotropins. Indeed, Kp has been used to safely and effectively stimulate oocyte maturation in women at high risk of developing ovarian hyperstimulation syndrome who were undergoing IVF treatment [29], and Kp-10 restored T to normal levels in men with diabetes mellitus [19].

On a cautionary note, previous reports have shown that stimulation with hypothalamic trophic hormones in patients with existing pituitary adenomas may in rare cases result in bleeding within the adenoma [30]. The infusion of Kp in the two patients described here was well tolerated. There was no episode suggestive of pituitary necrosis, either during or after Kp administration, as indicated by the absence of headache or visual disturbances. In addition, hypophyseal magnetic resonance imaging performed in both patients a few months after the infusion showed no evidence of adenoma necrosis or hemorrhage. Mean PRL levels remained elevated in both patients, suggesting that there was no adenomatous necrosis or hemorrhage. Overall, Kp-10 was well tolerated, and no adverse side effects were found in this study, as in previous studies using this dose regimen and treatment duration [17–20].

The findings in the current study suggest that the site of action of PRL is at the Kp neuron because an action at the GnRH neuron would have prevented or at least diminished GnRH/gonadotropin responses to Kp-10, whereas the LH and FSH responses were rapid and very substantial (four- to fivefold for LH, which is greater than LH responses to Kp-10 in healthy women) [20]. It is plausible, however, that PRL action is at the level of NKB secretion in humans, as we have shown robust stimulation of gonadotropins by Kp-10 in patients with NKB and NKB receptor−inactivating mutations [17] and in healthy women receiving an NKB antagonist [18, 31], suggesting that NKB action is upstream of Kp. Thus, if PRL inhibition of gonadotropins is at the level of NKB, this would result in decreased endogenous Kp secretion and a robust response to Kp-10, as we found in our patients. In our studies on mice rendered anovulatory by continuous PRL infusion, there was a decrease in Kp and GnRH messenger RNA and protein but no change in NKB [16], suggesting that the effect of PRL is not at the level of NKB, at least not in laboratory mice. It might be possible to tease out the level of effect of hPRL in humans by examining gonadotropin response to NKB agonists (*e.g.,* senktide) [18, 31]. If responses to senktide are sluggish, we could interpret that the site of action of PRL is at the level of Kp diminution; however, if the effect is robust, it would suggest that elevated PRL operates by inhibiting NKB.

Patient 1 exhibited a high LH/FSH ratio in contrast to patient 2, who had a higher FSH/LH ratio. This may be due to a higher GnRH pulse frequency in patient 1, which favored LH secretion [32]. Patient 1 had six LH pulses compared with three pulses in patient 2. Patient 1 appeared to have more advanced follicular development and a higher E2 level after Kp-10, which may account for higher pulse frequency resulting from positive feedback; however, further speculation is unjustified in a study with only two patients.

The elevation in gonadotropins by Kp-10 was accompanied by an increase in E2 and IB to levels similar to those found in the follicular phase of the menstrual cycle of healthy women. This suggests that Kp-10 induced the release of bioactive gonadotropins and that antral follicular development had been initiated in the two patients with hPRL-HA. Our results suggest the possibility of utilizing Kp-10 or analogues for induction of ovulation in women with elevated PRL levels from microadenomas that are unresponsive to dopamine agonists [27] and in women undergoing neuroleptic/antipsychotic therapy but desiring pregnancy and unable to tolerate antipsychotic drug withdrawal [33]. From a therapeutic viewpoint, Kp therapy in this setting may have advantages over alternative conventional treatments. GnRH therapy requires pulsatile administration of the medication by a portable pump, which is quite restrictive. Gonadotrophin administration requires tight ovarian monitoring to avoid ovarian hyperstimulation. Treatment with Kp is more physiological and potentially more convenient, as previous studies have shown that twice-daily subcutaneous injection is effective [28].

There is also evidence that E2 can have a protective effect in schizophrenic psychoses [30], thus suggesting that in patients with hPRL who are taking antipsychotics, elevation of E2 by Kp may benefit their mental state in addition to restoring fertility [33, 34].

In conclusion, this study demonstrated that Kp-10 can restore gonadotropin levels and ovarian function in women with hPRL-HA. The inhibitory effects of PRL on the gonadotrope axis appeared to be mediated through Kp-secreting neurons, as in rodents.
